# RETRACTION: A Rare Case of Tumefactive Demyelination of Brain: A Case Report and Literature Review

**DOI:** 10.1002/ccr3.73065

**Published:** 2026-07-03

## Abstract

**RETRACTION**: InbanP. , AkumaO. , ElaviaZ. , AkumaC. M. , SinghM. , KachhadiaM. P. , and MusiimeA. B. J. , "A Rare Case of Tumefactive Demyelination of Brain: A Case Report and Literature Review," Clinical Case Reports 11, no. 12 (2023): e8369. 10.1002/ccr3.8369.38130854 PMC10733795

The above article, published online on 21 December 2023 in Wiley Online Library (wileyonlinelibrary.com), has been retracted by agreement between the journal Editor‐in‐Chief, Dr. Roberto Berebichez‐Fridman; and John Wiley & Sons Ltd. A third party reported that the images in Figures 1 and 2 as well as the values in Table 1 had been copied and manipulated from an online presentation on social media without permission and consent from the original researchers, the original institution, or the patient. The original images were posted online prior to submission of this article.

The authors responded to an inquiry by the publisher and stated that the patient featured in the case report presented at Government Medical College and Hospital, Chennai, Tamil Nadu, India. The authors further declared that Dr. Preeti Kumari Yadav was the author who was primarily involved in direct clinical interaction with the patient. The authors supplied documents in order to confirm that the case presentation took place at this institution. However, their documentation was found to be insufficient and unverifiable.

The retraction has been agreed to because the evidence of image reuse indicates that the case report as presented includes fabricated and misrepresented data. The authors were informed about the retraction.

**RETRACTION**: P. Inban , O. Akuma , Z. Elavia , C. M. Akuma , M. Singh , M. P. Kachhadia , and A. B. J. Musiime , "A Rare Case of Tumefactive Demyelination of Brain: A Case Report and Literature Review," Clinical Case Reports 11, no. 12 (2023): e8369. 10.1002/ccr3.8369.38130854 PMC10733795

The above article, published online on 21 December 2023 in Wiley Online Library (wileyonlinelibrary.com), has been retracted by agreement between the journal Editor‐in‐Chief, Dr. Roberto Berebichez‐Fridman; and John Wiley & Sons Ltd. A third party reported that the images in Figures 1 and 2 as well as the values in Table 1 had been copied and manipulated from an online presentation on social media without permission and consent from the original researchers, the original institution, or the patient. The original images were posted online prior to submission of this article.

The authors responded to an inquiry by the publisher and stated that the patient featured in the case report presented at Government Medical College and Hospital, Chennai, Tamil Nadu, India. The authors further declared that Dr. Preeti Kumari Yadav was the author who was primarily involved in direct clinical interaction with the patient. The authors supplied documents in order to confirm that the case presentation took place at this institution. However, their documentation was found to be insufficient and unverifiable.

The retraction has been agreed to because the evidence of image reuse indicates that the case report as presented includes fabricated and misrepresented data. The authors were informed about the retraction.

